# Clinical study on concurrent use of electro-acupuncture or Chuna manual therapy with pregabalin for chemotherapy-induced peripheral neuropathy: safety and effectiveness (open-labeled, parallel, randomized controlled trial, assessor-blinded): A study protocol

**DOI:** 10.1097/MD.0000000000018830

**Published:** 2020-01-17

**Authors:** Jin-Hyun Lee, Tae jin Cho, Min Geun Park, Ji-Hoon Kim, Sung Kyu Song, Shin-Young Park, Yun-Young Sunwoo, Ilkyun Lee, Tae-Yong Park

**Affiliations:** a Institute for Integrative Medicine, Catholic Kwandong University International St. Mary's Hospital; b Department of Emergency Medicine; c Department of Surgery, Catholic Kwandong University International St. Mary's Hospital, Catholic Kwandong University College of Medicine; d Department of Surgery, Catholic University of Korea, Incheon St. Mary's Hospital; e Iksoodang Korean Medical Clinic, Incheon, South Korea.

**Keywords:** acupuncture, chemotherapy-induced peripheral neuropathy, electroacupuncture, manual medicine, pregabalin

## Abstract

**Introduction:**

Chemotherapy-induced peripheral neuropathy (CIPN) is one of the major side effects of chemotherapy. Its main symptoms are pain, paresthesia, and numbness. However, the mechanisms underlying the development of CIPN remain unclear and standard treatments have not been established. Recently, there has been a growing interest in various approaches to overcome the limitations of the existing treatments. This study aims to evaluate the efficacy and safety of the concurrent use of two complementary and alternative therapies: electroacupuncture (EA) and Chuna manual therapy (CMT), with pregabalin, which is the conventional pharmacotherapy for neuropathic pain.

**Methods/design:**

This is an open-label, parallel, assessor-blinded randomized controlled trial, which includes 90 patients with colorectal and breast cancer, who developed CIPN. After a 2-week preparation period, the patients are divided into three groups (pregabalin administration group, pregabalin + EA treatment group, and pregabalin + CMT treatment group), treated for approximately 5 weeks and followed-up 4 weeks after treatment. The primary outcome is assessed using the Functional Assessment of Cancer Therapy/Gynecologic Oncology Group Neurotoxicity subscale score (version 4.0) and the secondary outcome is measured using the Quality of Life Questionnaire-CIPN 20-Item Scale (version 3.0) and the quality of life questionnaire (version 3.0) developed by the European Organisation for Research and Treatment of Cancer. Moreover, exploratory efficacy and safety evaluations will be conducted based on the chemotherapy-completion rate and nerve conduction studies.

## 1 Introduction

Chemotherapy-induced peripheral neuropathy (CIPN) is a significantly common adverse effect of anticancer drug, with high prevalence. Approximately 68% of patients receiving chemotherapy develop symptoms of CIPN within 1 month,^[1]^ which include neuropathic pain, numbness, burning, and tingling of the skin. These symptoms may last for a long time, resulting in a rapid deterioration in the quality of life.^[2–4]^

Several anticancer agents can induce CIPN, including platinum analogs (cisplatin, carboplatin, and oxaliplatin), antitubulins (paclitaxel, docetaxel, ixabepilone, vincristine), proteasome inhibitors (bortezomib), and others (thalidomide); however, the mechanisms underlying this drug-induced neurotoxicity remain unclear, and genetic risk factors, past medical history, and association with other drugs are also known to be closely related to the occurrence of CIPN.^[5,6]^ Due to these limitations, there is no standardized treatment protocol for CIPN. In general, various drugs that are effective for neuropathic pain, such as nerve-protective agents, ion channel targeted agents, antioxidants, and anti-inflammatory agents are used for the treatment of CIPN, based on the clinician's preference and the patient's symptoms; however, the evidence of their efficacy for treating CIPN is insufficient, except duloxetine.^[7–9]^ Moreover, these drugs may also be less effective and causes adverse effects such as dizziness, weight gain, somnolence, peripheral edema, and fatigue.^[10,11]^

Recently, various studies have reported the treatment of CIPN with complementary and alternative medicine (CAM).^[12,13]^ Acupuncture (including electroacupuncture [EA]) is the most popular CAM therapy and is reportedly effective for treating cancer-related symptoms, such as CIPN, aromatase inhibitor-associated arthralgia, and post-neck dissection pain.^[14]^ Moreover, some articles on herbal medicine, manual medicine and exercises reported positive effects on several peripheral neuropathy, including CIPN.^[15,16]^ However, research has focused only on the efficacy of each CAM intervention for CIPN, and there are very few studies on its efficacy combined with conventional treatment.

The present study aims to verify the safety and efficacy of the concurrent use of EA or Chuna manual therapy (CMT) (a manual medicine treatment widely used in Korea) with pregabalin for patients with CIPN (especially, taxane-induced peripheral neuropathy in breast cancer and oxaliplatin-induced peripheral neuropathy in colorectal cancer), compared to pregabalin therapy alone. We hope that this study will validate the efficacy and safety of combination therapy and suggest a new approach for the treatment of CIPN.

## 2 Objective

The study aims to verify the hypothesis that the concurrent use of CMT or acupuncture treatment with pregabalin, a medication commonly used for CIPN is more effective and safe for the relief of CIPN symptoms than is pregabalin-alone therapy.

## 3 Methods

### 3.1 Trial registration

This study has been registered in the Clinical Research Information Service (CRIS; trial registration number: KCT0004217; trial protocol version: IS18ENSI0054 version 2.0; https://cris.nih.go.kr/cris/en/search/search_result_st01.jsp?seq=12752).

### 3.2 Study design

**Figure 1:**
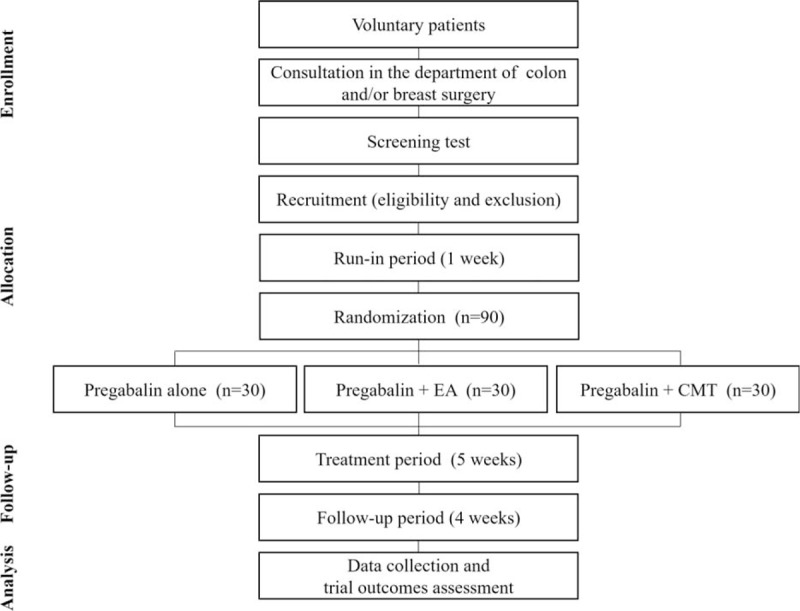
Flow chart of the trial. CMT = Chuna manual therapy, EA = electroacupuncture.

This study is designed as an open-label, parallel, assessor-blinded randomized controlled trial. This study will be conducted at the Catholic Kwandong University International St. Mary's Hospital, Incheon, South Korea. The diagrammatic representation of this study is presented in Figure 1. The patients will receive a full explanation of the details of the trial from investigators. Through this procedure, if they agree to participate in the trial, a signed consent form will be obtained.

The patients visit the hospital five times for evaluation. Screening (visit 1) includes only those participants who have submitted the informed consent form. For screening, demographic information, medical history, physical examination, vital sign, questionnaire survey, laboratory test, pregnancy test (childbearing age females), neurological exam (decided by the attending physician for exclusion diagnosis purpose), and selection/exclusion criteria were be evaluated. Patients who have passed the screening test will have a 7-day run-in period, during which all medications prescribed for controlling the symptoms of peripheral neuropathy will be stopped.

Ninety patients were enrolled and randomly divided into three groups. However, if no such drugs were being used during the screening test, randomization began immediately. The visits are designed for patient evaluation at approximately 2 weeks (visit 2: baseline visit), 4 weeks (visit 3), 7 weeks (visit 4), and 11 weeks (visit 5: outpatient follow up) after the screening test. These visits include evaluation of the medical and medication history, basic physical examination, vital signs, laboratory tests, pregnancy test (for women of childbearing age), primary effectiveness and secondary effectiveness assessments, exploratory effectiveness assessment (chemotherapy completion rate: whether all planned chemotherapy procedures were performed without interruption due to CIPN), and nerve conduction study (NCS). The coordinators of the trial will inform the patient of the next visit schedule for each visit and encourage participation. The clinical trial conductor will instruct the participant to ensure compliance with the treatment. The assessment of treatment compliance with pregabalin will be based on the planned dose and the actual dose taken, as well as the quantity returned and the quantity not returned at each patient visit. The calculation is based on the actual number of treatments compared to the planned number of treatments for EA and CMT.

Only those patients who have completed the informed consent form with full acceptance of the expected effects and predicted adverse reactions to clinical trial interventions prior to commencement of the study may participate in the study. In the event of an accident or adverse event, appropriate measures will be taken according to the investigator's judgment and the participant will be compensated. Moreover, the study will be conducted ethically, in compliance with the principles of the Good Clinical Practice (GCP) guidelines and the revised version of the Declaration of Helsinki.

### 3.3 Sample size calculation

This study is a pilot clinical trial, which aims to evaluate the efficacy and safety of combined treatment with EA and CMT, compared to the conventional-therapy group (pregabalin treatment group). We determined that it would be desirable to proceed with the minimum number of participants possible, to meet the goals of this study. Moreover, earlier clinical studies involving acupuncture or EA for CIPN have included sample sizes of about 20 participants per group.^[17,18]^ Thus, we concluded that this study should include 30 participants in each group (total sample size: 90). Even if the dropout rate is assumed to be 20%, this sample size is larger than that for similar earlier studies.

### 3.4 Intervention

#### 3.4.1 Pregabalin (usual care) group

Pregabalin is known to have positive effects on several symptoms of neuropathic pain, including CIPN.^[11]^ Pregabalin group with repeated oral administration of one capsule of pregabalin (Product name, Lyrica capsule, 75 mg) 30 min after meals twice a day after the screening test. If the participant is already taking more than 150 mg/day of pregabalin, the dose will be maintained without reduction (≤600 mg/day).

#### 3.4.2 Electroacupuncture treatment group

The EA-treatment group is to be treated with EA, in addition to the treatment for the conventional-therapy group. A disposable sterilized needle made of stainless steel (0.2 mm ∗ 30 mm, Spring Handle Needle, DONGBANG Medical Co, LTD) will be used for treatment, and the EA device is STN-330 (Maximum Output Frequency: 240 Hz, Maximum Output Current-Low 7.3 mA, High 13.0 mA, Stratek. Co. LTD). The acupuncture points to be used for treatment are: ST 40, GB 34, EX-LE 10, EX-UE 11, TE 5, LI 4, ST 36, KI 6, CV 4. Of them, ST 40 and GB 34, TE 5; LI 4, ST 36, and KI 6 are paired with each other and a 20-min long electrical stimulation is applied with a 2-Hz mixed pulse, while EX-LE 10, EX-UE 11, and CV4 are not electrically stimulated. The final acupoints were selected through network analysis for the EA treatment. The EA treatment is conducted three times per week for the first 2 weeks and then twice per week for the following 3 weeks.

#### 3.4.3 Chuna manual therapy group

The CMT group is treated with CMT, in addition to conventional therapy. In Chuna treatment, patients with colorectal cancer lie down on their side and Sunwoo manual therapy, which helps joint movement and improves overall function is used.^[19]^ Sunwoo manual therapy may cause unnecessary irritation to the area affected by breast cancer. Thus, CMT for meridian sinew system, which is applied to the nerves of the ribs, upper and lower limbs is utilized. CMT is conducted twice a week for the first 2 weeks and once a week for the following 3 weeks.

### 3.5 Inclusion and exclusion criteria

#### 3.5.1 Inclusion criteria

The inclusion criteria as follows:

1. adult men and women aged 18 to 85 years;
2. patients with colorectal cancer with oxaliplatin-induced peripheral neuropathy or patients with breast cancer patients with taxane-induced with peripheral neuropathy (provided that the patients have had peripheral neuropathy for 1 month or longer after the chemotherapy before the time of screening and have peripheral grade 2 or higher sensory/motor neuropathy, based on the Common Terminology Criteria for Adverse Events (CTCAE) V5.0 at the time of screening);
3. those who do not have significant restrictions on their behavior and life, corresponding to Eastern Cooperative Oncology Group (ECOG) 0–2;
4. patients who can read the symptom questionnaire, comprehend it, and answer accordingly;
5. patients who have given their consent for the clinical protocol and follow-up and in the Institutional Review Board (IRB)-approved document.

#### 3.5.2 Exclusion criteria

The following patients would be excluded from participating in the trial:

1. patients with neuropathy, caused by factors besides chemotherapy (diabetes, peripheral vascular disease, alcohol-induced neuropathy, drug-induced neuropathy, and other neurological disorders);
2. patients with a history of renal disease or with a medical history that contraindicates the use of pregabalin, according to the treating physician;
3. patients with conditions that affect the progress of Korean medicine treatments (EA, CMT) such as skin lesion or fractures;
4. patient with fear or aversion toward Korean medicine (EA therapy, CMT);
5. patients who received oriental medical treatment such as acupuncture, moxibustion, cupping therapy, and oriental medication for CIPN, within 1 week of screening;
6. patients with other diseases or factors that may be regarded as inappropriate for the clinical study;
7. if the treating physician has determined that the patient is unsuitable for participation in this study;
8. women of childbearing age and pregnant or lactating women; and
9. those who participated in other clinical studies within 30 days prior to the screening for this clinical study.

### 3.6 Recruitment, randomization, blinding and non-blinding

Advertisements for patient recruitment will be posted on the Catholic Kwandong University International St. Mary's Hospital bulletin boards, along with posters, banner advertisements and internet cafes. All advertisements will be run after IRB approval.

Individuals who have given written consent for participating in the study will be assigned a screening number, in order of the outpatient visit at screening. Unblinded sub-investigators, who do not influence the research results and analysis, will refer to the randomization list that has been prepared by a commissioned professional statistician, and assign participation numbers by stratification (based on the presence of breast cancer or colorectal cancer). The randomized list will be generated by layer considering the stratification factor by the block randomization method, and participants will be allocated to the pregabalin group, the pregabalin + EA group, and the pregabalin + CMT group in a ratio of 1: 1: 1. A randomization table is generated by an independent statistician and patients are assigned to one of three groups according to the randomization table. Screening numbers, randomization numbers, and initials assigned to each participant are used as subject identification codes to identify the participant until the end of the clinical trial.

The pregabalin used in this study is not a clinical investigational product and does not require allocation concealment, since it is administered to all groups. Moreover, it is impossible to blind the investigator and the participant in each group due to the nature of the study intervention, which is based on the contact between the patient and the medical staff. Instead, the investigator maintains blinding to prevent bias in evaluating the participant. If the clinical trial is completed, all clinical report forms (CRFs) are collected, the database process is terminated, and unblinding will be performed, if required for statistical analysis. Moreover, if it is deemed necessary to confirm the subject group due to an emergency situation during the clinical trial, the randomization code will be disclosed only to the participant, according to the principal investigator's discretion. If it is not possible to consult with the principal investigator and the situation is urgent, the randomization code of the subject will be disclosed after consultation with the randomization code officer. If unblinding is performed, the documentation of the reason for the unblinding will be retained and the participant with the disclosed randomization code will not be able to continue the trial.

### 3.7 Rescue therapy and concomitant medications

If a participant experiences unbearable pain at the screening visit, he/she may be allowed to take acetaminophen. If the participant takes any rescue medication, the date and dose of administration will be recorded in the study participant's log. The dose of rescue medications should not exceed 4000 mg/day and should not be taken within 8 h prior to the participant's log entry for every morning. Moreover, medication is permitted in the following cases:

1. continuous low-dose aspirin use that was prescribed for non-analgesic purposes at a fixed dose 1 month before the run-in period (≤375 mg/day), hypoglycemic agents, and hypertension drugs (however, the dose taken or the regimen at the start of the study of the low-dose aspirin does not change throughout the clinical trial);
2. medications that were being taken by participants at the time of screening and would not affect the results of this clinical trial;
3. the administration of the drugs used for the transient treatment of other disorders will be determined at the discretion of the principal investigator (or sub-investigator or attending physician).

If concomitant medication is administered during the trial period, the name of the drug, the daily dose, and the duration of administration should be noted on the case report form.

### 3.8 Study outcomes

#### 3.8.1 Primary outcome

The primary outcome in this study measures the changes in the Functional Assessment of Cancer Therapy/Gynecologic Oncology Group Neurotoxicity (FACT/GOG-Ntx) subscale (version 4.0, Korean) in comparison with the baseline, in which scores from 11 questions, each consisting of 0 to 4 points will be evaluated. A higher score indicates a more severe degree of peripheral neuropathy.^[20,21]^ The FACT/GOG-NTX questionnaire is to be self-administered by the participants.

#### 3.8.2 Secondary outcome

Secondary outcome assessment measures the change and rate of change in the Quality of Life Questionnaire-CIPN 20-Item Scale (EORTC QLQ-CIPN 20) and Quality of Life questionnaire (EORTC QLQ-C30, version 3.0) developed by the European Organisation for Research and Treatment of Cancer in comparison to the baseline.^[22–24]^ EORTC QLQ-CIPN 20 consists of 20 questions, with 9 sensory nerve-related questions, 8 motor neuron-related questions, and 3 autonomic nerve-related questions. A higher score indicates a more severe degree of peripheral neuropathy.^[22]^ EORTC QLQ-C30 is a tool for measuring health-related quality of life and consists of 2 items of the overall functional state, 15 items of function, and 13 items of symptoms. A lower overall score indicates a better quality of life.^[25]^

#### 3.8.3 Exploratory efficacy

Exploratory efficacy evaluation is based on the chemotherapy completion rate and NCS. The chemotherapy completion rate is used for patients enrolled in clinical studies where chemotherapy is in progress and is calculated as follows: chemotherapy completion rate = number of chemotherapy sessions completed after research project/chemotherapy sessions planned in the future∗100. The time point of investigating the chemotherapy completion rate is when all scheduled chemotherapy schedules are completed. NCS is performed as a statistical selective test and is conducted in 1 to 2 of 10 patients. The visit-window of the NCS that should be performed during the baseline visit will be ± 4 days.

#### 3.8.4 Safety and adverse events monitoring

Safety assessment includes:

1. basic physical examination, vital signs, and electrocardiogram;
2. examination of the presence of adverse reactions;
3. clinical laboratory testing; and
4. neurological tests.

The sub-investigator will record the details of any adverse reactions and use of concomitant medications during the clinical trial in the case report form. In case of an adverse reaction, the symptoms and signs of the adverse reaction, duration (start date/end date), severity, result, significance, causal relationship with the clinical investigational drug, and measures taken for its alleviation will be recorded. The names, dosages, duration of administration, reason for administration, etc, of the concomitant medications will be recorded in detail.

Clinical laboratory tests will be performed at visits 1, 2, 3, and 4. Since this study will evaluate the efficacy and stability of the combination of pregabalin, EA, and, CMT, it differs from clinical studies evaluating the safety and pharmacokinetic properties of drugs. Therefore, this study conducts clinical laboratory tests for the following items for the purpose of the standard safety lab test:

1. hematology: hemoglobin, hematocrit, red blood cell count, white blood cell count and differential count, platelet count;
2. serum chemistry: glucose, aspartate aminotransferase (AST), alanine aminotransferase (ALT), alkaline phosphatase, creatinine phosphokinase (CPK), uric acid, total bilirubin, blood urea nitrogen (BUN), electrolytes (sodium, potassium, chloride, calcium, phosphorus), total protein, albumin, serum creatinine (SCr);
3. coagulation: prothrombin time, activated partial thromboplastin time (aPTT);
4. urinanalysis: pH, protein (albumin), glucose, leukocyte esterase, blood;
5. pregnancy test (for women of childbearing age)

#### 3.8.5 Early termination or dropout

The early termination or dropout criteria are as follows:

1. if the participant has been administered a drug that is expected to affect the safety or efficacy of the investigational drug including neuropathic pain medications; antiepileptics and antidepressants, excluding pregabalin (concomitant treatment with selective serotonin reuptake inhibitors [SSRI] and tricyclic antidepressants [TCA] is allowed only when the antidepressants are used in stable administration conditions, without change in the type, dose, and usage from 14 days before the run-in period); herbal therapies besides those included in the present study, which may affect the outcome of this clinical trial; exercise therapy; physiotherapy; anti-anxiety agents; sleeping pills (all sleeping pills other than triazolam, zopiclone, and zolpidem tartrate); anti-anxiety agents; analgesics; muscle relaxants; steroids; cilostazol; prostaglandins and related drugs; local anesthetics; N-methyl-D-aspartate receptor (NMDA) antagonists; sodium channel blockers; central sympathetic neurosuppressants; vitamin B1 and/or B12 prescribed for peripheral neuropathic pain; drugs that may cause irreversible retinal degeneration; and other investigational drugs
2. if the participant requests the discontinuation of clinical trial treatment or withdraws consent
3. if a serious adverse event has occurred and the investigator determines that the trial cannot be continued
4. if serious violations of the protocol, including inclusion/exclusion criteria, are newly discovered during clinical trials
5. if treatment compliance is below 70% and
6. if, for any other reason, the principal investigator/sub-investigator determines that the trial should be discontinued.

#### 3.8.6 Data collection, access, and management

All information regarding the patient is anonymized through initial processing, and all investigators are obliged to maintain confidentiality of the results. The source document is registered immediately when the data are collected, and it will be recorded in the CRF. All the documents of trial will be kept safe, and only those who have been approved by the principal investigator will have access to all data related with the trial.

Data management of this clinical trial will be conducted in accordance with the standard working guidelines of the Catholic Kwandong University Clinical Research Center, and other matters not specified in the protocol will be conducted following the International Council for Harmonisation of Technical Requirements for Pharmaceuticals for Human Use (ICH) guideline for GCP and Korea-GCP standards.

### 3.9 Statistical analysis

All statistical analyses will be conducted by a blinded professional statistician. All participants who provided written consent and were assigned a screening number for the study are included in the screening set, and patients randomized to the treatment group are included in the randomized set. Intention-to-treat (ITT) analysis will be performed in patients who have received at least one pregabalin trial or at least one session of acupuncture or CMT. Per-Protocol Set (PPS) analysis will be performed in patients who meet the inclusion/exclusion criteria, have no significant conditions affecting the efficacy assessment, show > 80% treatment compliance, and have performed at least one evaluation after the baseline evaluation.

The statistical analysis for the efficacy evaluation, based on ITT analysis and PPS analysis are conducted in parallel. All statistical tests will be performed as two-sided tests with a significance level of 5%, and two-sided 95% confidence intervals. Primary outcome assessments are performed with ANOVA analysis to test the differences between the groups for changes in FACT/GOG-NTX at the end of week 5, compared to baseline. If the differences between groups are significant, the Tukey method will be used. For secondary outcome evaluation, continuous variables are analyzed with ANOVA analysis or Kruskal–Wallis test, and categorical variables are analyzed with Pearson's chi-squares test or Fisher's exact test. The Exploratory Efficacy assessment will summarize the descriptive statistics on the chemotherapy completion rate, followed by the appropriate statistical analysis.

For the occurrence of adverse events, the number of cases, number of participants, severity, and causal relationship with the investigational drug can be statistically analyzed by group, and nonparametric methods can be applied as needed. The results of vital signs, physical examination, electrocardiograms, and laboratory tests will be reviewed in a comprehensive manner, and statistical analysis will be conducted as necessary for the test items deemed clinically significant by the investigators. Depending on the nature of the data, continuous data will be summarized by observed values, mean, standard deviation, median, minimum, and maximum for each visit, and categorical variables will be summarized by frequency and percentage.

#### 3.9.1 Quality control and data monitoring

This study will be monitored by CROCENT Co., Ltd., a CRO company that consults with institutions conducting clinical trials to ensure compliance with protocol and K-GCP. During monitoring, crosschecks will be made with the evidence to ensure that the documents (trial master files, CRF, informed consent forms, and adverse events reports) are complete and clear.

### 3.10 Ethics and dissemination

#### 3.10.1 Research ethics approval

This trial has received complete ethical approval from the Ethics Committee of Catholic Kwandong University International St. Mary's Hospital (IS18ENSI0054).

#### 3.10.2 Protocol amendments

The investigators who want to protocol amendments should first discuss it with the principle investigator, and they can change the protocol after obtaining approval from the IRB. However, when a dangerous situation occurs and immediate care is needed, the protocol change will be reported to the IRB at a later time.

#### 3.10.3 Post-trial care

If the patients experience unexpected accidents or injuries, the patients will receive appropriate medical care at the Catholic Kwandong University International, St. Mary's Hospital. Additionally appropriate compensation will be made by the insurance company, according to the patient compensation rules of the trial.

#### 3.10.4 Declaration of interests

This study was supported by the Traditional Korean Medicine R&D program funded by the Ministry of Health and Welfare through the Korea Health Industry Development Institute (KHIDI) (Grant number: HI18C1935).

#### 3.10.5 Competing interests

None.

## 4 Discussion

Through sampling analysis of Health Insurance Review and Assessment Service (HIRA) data on Korean medicine (∼1 million persons, conducted on January 15, 2018), the analysis on prescription frequency of CIPN as primary disease and secondary disease for 10 years showed no reported case, indicating that currently, there has been no approaches in terms of Korean medicine treatment of CIPN in the corresponding clinical studies.^[26]^

Korea has the world's highest incidence of colorectal cancer. In addition, the number of breast cancer patients in Korea increased four times from 3801 in 1996 to 16,398 in 2010 and is in the trend of steady increase.^[27]^ Especially, colorectal cancer and breast cancer have high incidence rates and have a long treatment period compared to other types of cancers, during which the patients can experience multiple side effects of chemotherapy including CIPN.

This study will be conducted to evaluate the efficacy and stability of combining conventional treatment and complementary alternative treatment (oriental medicine) for CIPN induced by anticancer drugs used for patients with colorectal cancer and breast cancer based on various evaluation indices. We believe that this study will be instrumental in providing better alternative solutions to CIPN, a refractory disease and overcome the limitations of existing studies, despite its own limitations.

## Author contributions

Jin-Hyun Lee and Tae jin Cho contributed to the trial design and writing the manuscript. Ilkyun Lee, Min Geun Park, Ji-Hoon Kim, Sung Kyu Song, Shin-Young Park, and Yun-Young Sunwoo provided perspective on the selection of the trial interventions and advice on the trial procedure. Tae-Yong Park is responsible for writing the manuscript and managing and supervising the clinical trial. All authors have read and approved the final version of the manuscript.

Tae-Yong Park: 0000-0002-6803-5483.
